# A Rare Case of Endoscopic Removal of 63 Coins From the Stomach of an Adult

**DOI:** 10.7759/cureus.42599

**Published:** 2023-07-28

**Authors:** Vivek Saini, Abhishek Yadav, Sunil K Dadhich, Bobby Mitrolia, Rajendra Bhati

**Affiliations:** 1 Gastroenterology, Dr. Sampurnanand Medical College, Jodhpur, IND; 2 Medicine, Maulana Azad Medical College, Lok Nayak Hospital, Delhi, IND

**Keywords:** 63 coins from the stomach, dr. sampurnanand medical college, july 2022, jodhpur, endoscopic removal

## Abstract

The paper focuses on the use of endoscopy in the extraction of 63 coins from the stomach of an adult psychiatric patient. So far, most such cases were dealt with by traditional surgery, and endoscopy was used for the removal of a few coins only. The present work emphasizes that endoscopy is a better option than surgical intervention as it is faster and has a shorter recovery time, lower risk of infection, and lower cost.

## Introduction

Ingestion of coins, marbles, button batteries, and other inanimate objects made of plastic, glass, wood, and metal and of different shapes and sizes is common in children and uncommon in adults [1-8]. Small, blunt, round objects generally pass out along with feces without much problem and hence remain unreported. However, long, narrow, wide, and sharp-edged metallic objects are capable of perforation, impaction, bleeding, and infections, which cause discomfort and abdominal pain, forcing patients to the hospital [6]. While endoscopy is the preferred mode of removing up to five small objects, surgical removal is chosen for larger and sharper objects [1]. Of these, Indian coins are composed of stainless steel, although their dimensions exhibit slight variations depending on the year of minting. The diameter ranges from 25 mm to 22 mm, while the thickness measures around 1.5 mm. A case of surgical removal of 187 coins from the stomach of an adult at S. Nijalingappa Medical College, Bagalkot, India, was reported in Hindustan Times on November 27, 2022, but no published literature on the extraction of a large number of coins by endoscopy exists. Hence, we report this rare and perhaps the maiden case of endoscopic removal of 63 coins from the stomach of a psychiatric patient in the Department of Gastroenterology, Dr. Sampurnanand Medical College, Jodhpur, India.

## Case presentation

**Figure 1:**
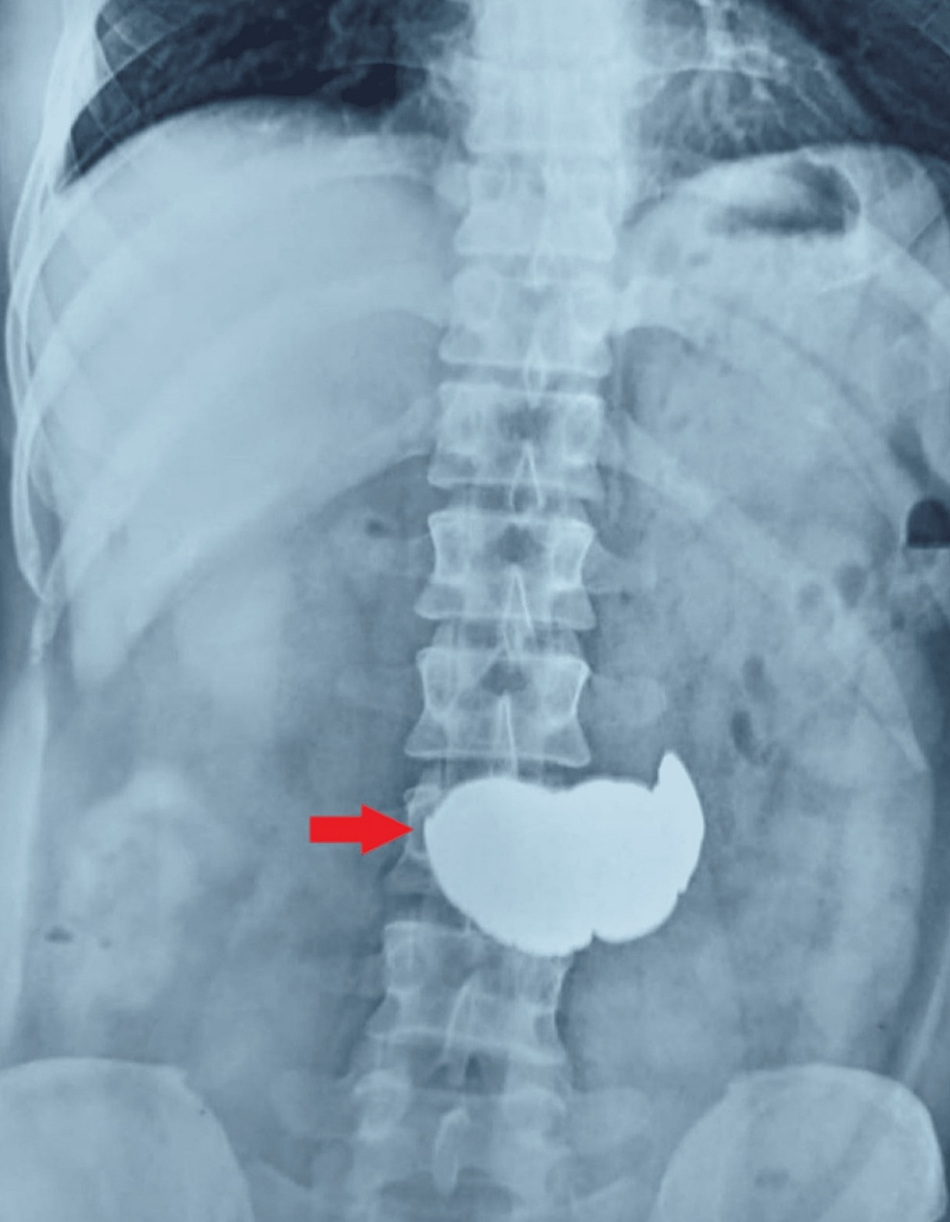
Abdominal X-ray showing heaped-up coins (red arrow) in the stomach

**Figure 2:**
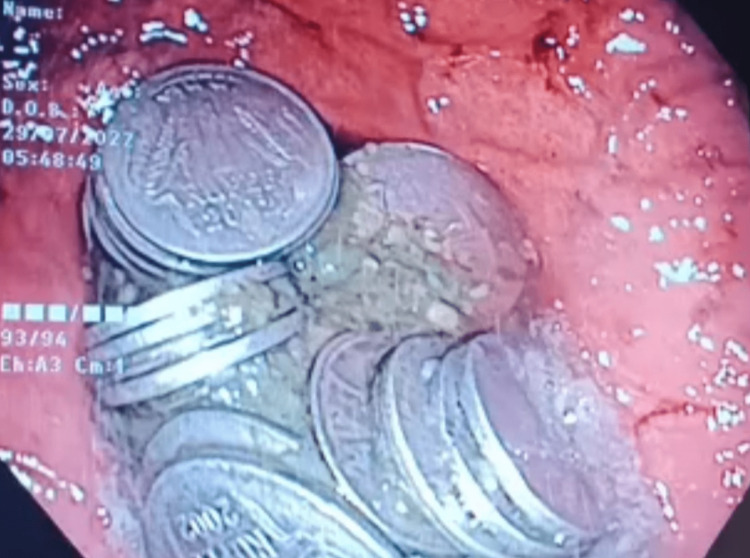
Endoscopic image of the stomach showing the stack of coins

**Figure 3:**
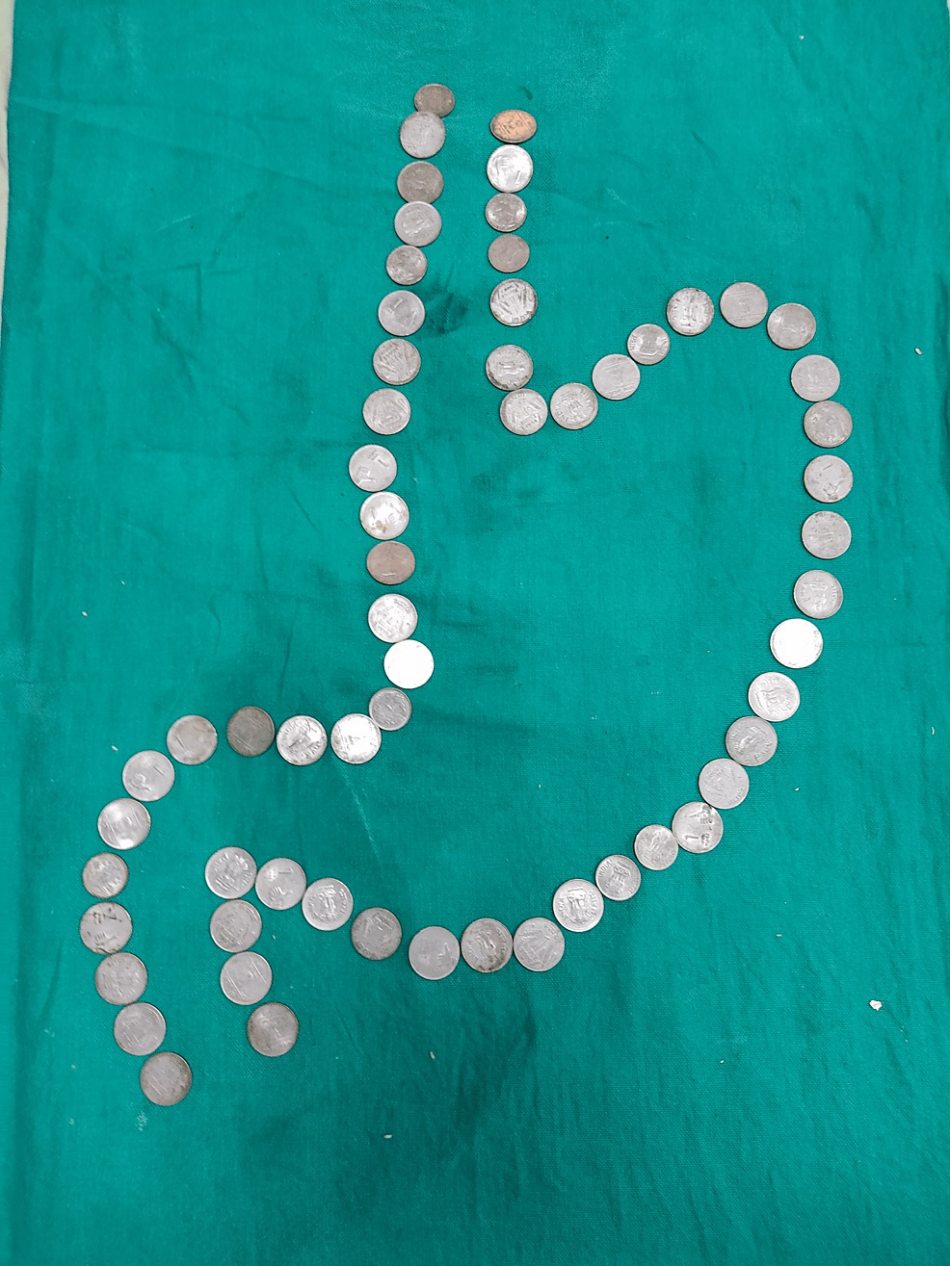
Extracted coins from the stomach

A 36-year-old male presented with complaints of severe abdominal pain on July 28, 2022. He was a known case of depression for the past two years and had been off antidepressants for two months. According to the history provided by his mother, the patient had been swallowing coins over the past few days. The pain was mainly in the upper abdomen and was associated with nausea and early satiety. However, there were no clinical features suggestive of obstruction, gastrointestinal (GI) bleeding, or perforation/peritonitis. The patient had stable vitals with a Glasgow Coma Scale (GCS) of 15/15. On examination, tenderness with an underlying hard mass was felt in the left upper quadrant. An X-ray of the abdomen confirmed the presence of a metallic lump in the stomach (Figure 1). The X-ray did not reveal any evidence of intestinal obstruction or perforation. Considering the alleged history of ingesting a large number of coins and its apparent complications, with the distant possibility of one or two button batteries in between the coins, it was planned to endoscopically remove the metallic objects. Mild sedation to counter patient anxiety was given in the form of IV midazolam by an anesthetist. During endoscopy, a stack of coins was observed in the fundus of the stomach (Figure 2). There was also diffuse erythema with erosions seen in the entire gastric mucosa; however, no active bleed or ulcerations were documented. After the initial berry picking of coins, it was planned to remove stacks of coins using the Roth Net Retrieval Basket (RotoNet Retrieval Net, Cook Medical, Bloomington, IN, USA). Each stack ranged between two and six coins. Due precautions to prevent mucosal injury were taken in the form of high air insufflation, keeping the basket containing stacks of coins in close proximity to the endoscope in the visual field, and waiting for spontaneous relaxation of the gastroesophageal junction and cricopharynx while maneuvering through these junctures. Extraction was done in two sessions as the patient had discomfort in keeping the jaw open for a long duration. A total of 63 coins were extracted, and the entire process took nearly two hours (Figure 3). There was no iatrogenic bleeding or perforation. The patient was hemodynamically stable throughout the procedure with no systemic adverse events encountered. Soon after the completion of the procedure, the patient was discharged.

## Discussion

Most of the published records pertain to 1-3 coins removed from the esophagus and stomach of children (Table 1) [7-14]. Button batteries are another type of object that necessitates immediate extraction due to the potential risk of mucosal injury from the electric current they generate. Moreover, if the battery leaks inside the gastrointestinal tract, it can cause corrosive injury. Most such patients have complaints of vomiting and abdominal pain. Their physical examination is often normal but crucial to exclude perforation, as evidenced by fever, tachycardia, peritonitis, subcutaneous crepitus, and swelling of the neck or chest [3]. X-ray is a customary diagnostic tool to determine the location, shape, and nature of the object. Sometimes, additional imaging tests are also required.

**Table 1:** Compilation of some important cases of coin extraction from the gastrointestinal tract

Case report	Age (year)/sex	Number of coins	Location of coins	Ingestion time/duration in the body	Symptoms/complication	Procedure adopted
Assiri et al. (2022) [7]	5/male	1	Upper esophagus	4 years	Pain, odynophagia, dysphagia, drooling	Surgical
Somani et al. (2009) [8]	12/male	1	Near ileocecal valve	7 days	Pain, vomiting, edematous ileocecal valve	Colonoscopy
Kim et al. (1999) [9]	22/female	1	Near ileocecal valve	3 weeks	Pain, fibrosis	Surgery
Pugh et al. (2021) [10]	3.6/male	1	Fundus	30 minutes	Vomiting, choking, pain	Endoscopy
T-Ping et al. (2006) [11]	1.7, 3.5, 4.7/male	1 in each	Upper esophagus	-	-	Intubation with rigid esophagoscopy and forceps, straight laryngoscope
Naidoo et al. (2004) [12]	3	1	Esophagus	2 months	Esophageal perforation	Right thoracotomy
Hussain et al. (2022) [13]	16/female	1	Upper esophagus	Few hours	Hypoactivity and drooling	Esophagoscopy
Arora et al. (2020) [14]	3/female	2	Esophagus	12-14 hours	Vomiting, dysphagia	Esophagoscopy

The course of treatment depends upon the location, size, and shape of the object. Items wider than 2 cm tend to remain in the stomach, while those longer than 5 cm are unable to pass the duodenum [4]. Coins typically tend to stay in the upper third portion of the esophagus, whereas approximately one-fourth of them make their way into the stomach. The ingested articles may lead to complications such as peritonitis, abscess formation, inflammatory mass formation, obstruction, fistulae, hemorrhage, or even death. Of these, coins can cause mucosal erosion and esophageal perforation [5].

**Figure 4:**
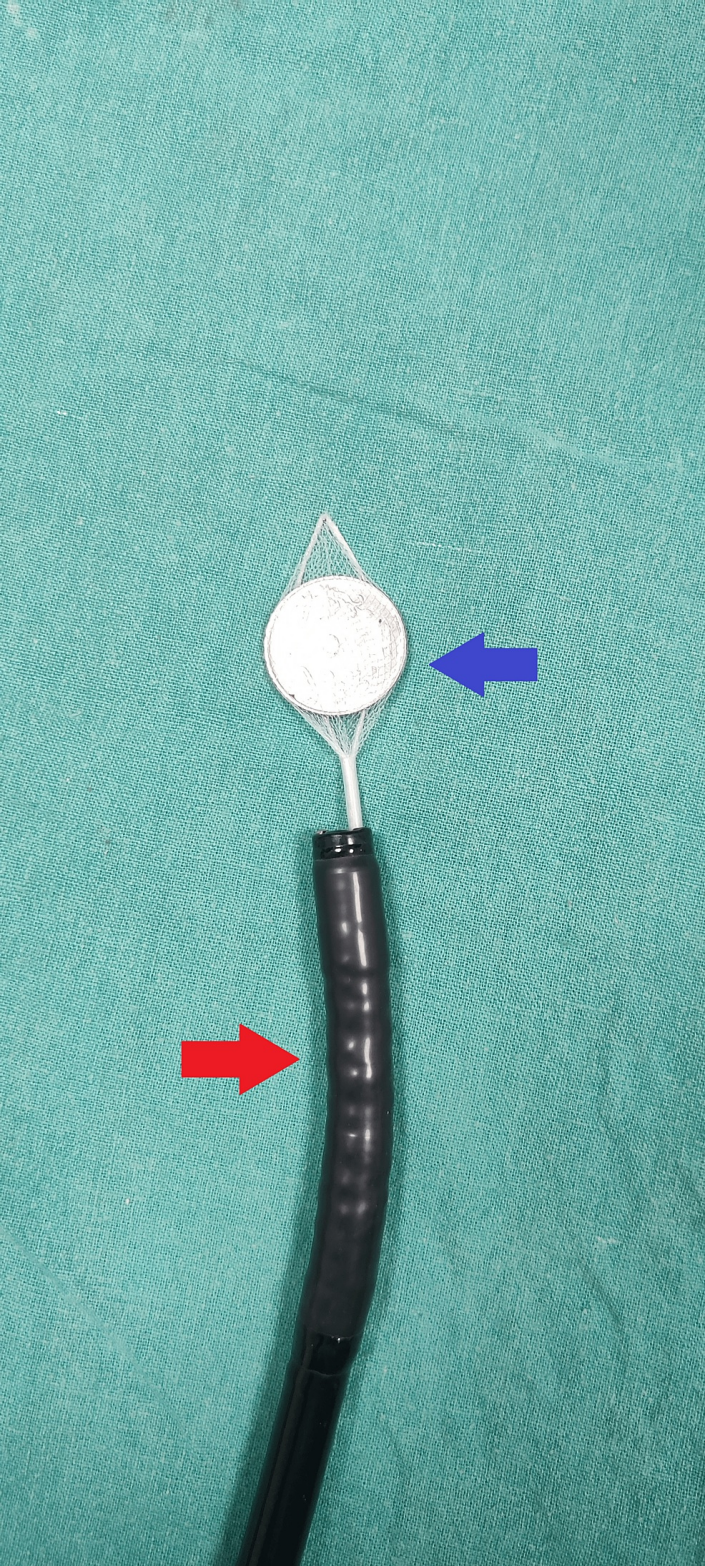
An illustration of an endoscope (red arrow) with a coin trapped in the mesh of the Roth Net Retrieval Basket (blue arrow)

In management, most clinicians wait for the natural elimination of the coin [13]. In case of failure, the European Society of Gastrointestinal Endoscopy recommends removal within 24 hours for sharp objects and 72 hours for blunt objects by endoscopy [3,4]. Coins in the esophagus need removal within 24 hours, which can be done by video endoscopes, straight laryngoscope along with long forceps, or using rigid esophagoscopes while the patient is intubated under anesthesia [6,7]. A coin in the stomach can pass in 4-5 days; if not, then in asymptomatic cases, a 2-4 week wait can be considered, as further delay may be risky [7]. However, in our case, due to the innumerable count of coins, endoscopic removal was planned on an urgent basis. Endoscopic removal could be done using polypectomy snares, Dormia baskets, Roth Net Retrieval Basket (Figure 4), or strong-toothed graspers. Sharp objects need special care, where retrieval forceps (rat tooth or alligator jaw grasping forceps) perform satisfactorily [6]. The present case was unique on account of (i) an innumerable number of coins, (ii) an unknown duration of ingestion, and (iii) little and unconfirmed feedback from the patient. Coin ingestion in adults is quite rare. Therefore, considering the apparent complications arising due to the above conditions and the suspected presence of button batteries with the coins, we decided on endoscopic extraction without delay.

## Conclusions

Endoscopic removal holds an edge over surgery in terms of fewer complications, short recovery time, and cost-effectiveness. However, its utility is limited to the upper GI tract. Here, we demonstrate the first case of successfully extracting 63 coins from the stomach using endoscopy, without any complications.
